# Electroacupuncture Inhibits the Activation of p38MAPK in the Central Descending Facilitatory Pathway in Rats with Inflammatory Pain

**DOI:** 10.1155/2017/7531060

**Published:** 2017-11-22

**Authors:** Man-Li Hu, Fei-Yan Zhou, Jing-Jing Liu, Yi Ding, Ju-Ming Zhong, Ming-Xing Ding

**Affiliations:** ^1^College of Veterinary Medicine, Huazhong Agricultural University, Wuhan 430070, China; ^2^College of Veterinary Medicine, Auburn University, 212 Greene Hall, Auburn, AL 36849-5518, USA

## Abstract

The mitogen-activated protein kinases (MAPKs), especially p38MAPK, play a pivotal role in chronic pain. Electroacupuncture (EA) relieves inflammatory pain underlying the descending pathway, that is, the periaqueductal gray (PAG), the rostral ventromedial medulla (RVM), and the spinal cord dorsal horn (SCDH). However, whether EA antagonizes inflammatory pain through regulation of p38MAPK in this descending facilitatory pathway is unclear. Complete Freund's adjuvant (CFA) was injected into the hind paw of rats to establish inflammatory pain model. EA was administrated for 30 min at Zusanli and Kunlun acupoints at 0.5, 24.5, 48.5, and 72.5 h, respectively. The paw withdrawal threshold (PWT), paw edema, and Phosphor-p38MAPK-Immunoreactivity (p-p38MAPK-IR) cells were measured before (0 h) and at 1, 3, 5, 7, 25, and 73 h after CFA or saline injection. EA increased PWT at 1, 3, 25, and 73 h and inhibited paw edema at 25 and 73 h after CFA injection. Moreover, the increasing number of p-p38MAPK-IR cells which was induced by CFA was suppressed by EA stimulation in PAG and RVM at 3 and 5 h and in SCDH at 5, 7, 25, and 73 h. These results suggest that EA suppresses inflammation-induced hyperalgesia probably through inhibiting p38MAPK activation in the descending facilitatory pathway.

## 1. Introduction

Chronic pain is maintained and modulated through nociceptive processes at spinal and supraspinal level. Electrophysiological and pharmacological researches have confirmed that stimulation of the periaqueductal gray (PAG) or the rostral ventromedial medulla (RVM) can influence spinal nociceptive process via inhibiting or facilitating nociceptive input [1–4]. Moreover, increased activity of peripheral nociception caused spinal sensitization and enhanced sensory information arriving at PAG and RVM, resulting in activation of descending facilitatory pathway [5–8]. Numerous studies document that the descending facilitation contributes to chronic pain states and maintenance of hyperalgesia [5, 7, 9, 10]. Further, blocking the descending facilitatory pathway attenuates chronic pain and hyperalgesia [5, 11, 12].

The activation of p38 mitogen-activated protein kinases (p38MAPK) signaling pathway plays a vital role in intracellular signal transduction on chronic pain [13–15]. Ni et al. [16] found that chronic constriction nerve injury induced mechanical hyperalgesia and increased expression of phosphorylated p38MAPK (p-p38MAPK) in the ventrolateral periaqueductal gray (vlPAG). Complete Freund's adjuvant (CFA) can induce inflammation pain and increase the number of Phosphor-p38MAPK-Immunoreactivity (p-p38MAPK-IR) cells in the RVM [17]. Studies have shown that p-p38MAPK promoting the transcription factors (e.g., tumor necrosis factor *α*, the activating transcription factor-2) and inflammatory mediators (e.g., interleukin-1 and cyclooxygenase-2) in the spinal cord could result in pain facilitation [18, 19]. Nevertheless, intrathecal injection or RVM microinjection of p38MAPK inhibitors decreases p38MAPK downstream propainful factors and alleviates hyperalgesia [9, 20, 21], suggesting that inhibition of p38MAPK activation may attenuate inflammatory pain underlying the descending facilitatory pathway.

Electroacupuncture (EA) is a traditional complementary and alternative medicine approach with the advantages of safety, efficiency, and quantifiability to ameliorate inflammatory pain [22, 23]. EA exerts effects against inflammatory pain, which has been confirmed by numerous clinical observations and studies [24–27]. Liang et al. reported that EA at bilateral Zusanli (ST36) and Kunlun (BL60) acupoints inhibited inflammatory-induced activation of spinal p38MAPK [28, 29]. However, whether EA antagonizes inflammatory pain through regulation of p38MAPK in the supraspinal levels is unclear.

In the present study, CFA-induced inflammatory pain model was applied to observe the effect of EA on the paw withdrawal threshold (PWT), paw volume, and the expression level of p-p38MAPK in PAG, RVM, and the spinal cord dorsal horn (SCDH). We hypothesize that EA could downregulate p-p38MAPK expression in the descending facilitatory pathway and then exert its inhibition function in the inflammatory pain.

## 2. Materials and Methods

### 2.1. Animal Preparation

Male Sprague-Dawley rats weighing 220 ± 20 g were provided by Hubei Provincial Center for Disease Control and Prevention (number 42000600005600). One hundred and forty-four rats were housed six per cage with food pellets and water ad libitum and maintained on 12 h alternate light–dark cycles (7 a.m. to 7 p.m.). A quiet environment was provided, and the room temperature was maintained at 22 ± 2°C. Experimental animals were accustomed to being approached, acclimatized in individual plastic enclosures on a metal mesh and housing facilities (30 min/day) for one week before experiment. The experimental protocol was approved by the Animal Care Center, College of Veterinary Medicine, Huazhong Agricultural University (Wuhan, China).

### 2.2. Establishment of Inflammation Model and Experimental Groups

**Figure 1:**
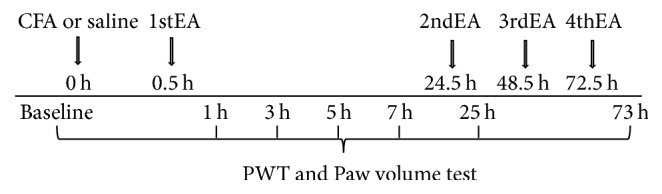
The timeline for the complete Freund adjuvant or saline injection, electroacupuncture treatment, the paw withdrawal threshold, and edema of the hind paw test.

Inflammatory pain model was induced by injection of CFA (Sigma, USA) into the plantar surface of left hind paw. The rats were separated randomly into 4 groups (36 rats/group): (1) the saline group with saline injection (100 *μ*L saline/per rat); (2) the CFA group with CFA injection (100 *μ*L CFA/per rat); (3) the CFA + EA group with CFA injection (100 *μ*L CFA/per rat) and EA treatment; (4) the CFA + sham group with CFA injection (100 *μ*L CFA/per rat) and needle insertion without electrical stimulation. EA treatment was given at 0.5, 24.5, 48.5, and 72.5 h after CFA or saline injection (Figure 1). To eliminate the stress effect, rats in saline group, CFA group, and CFA + sham group were immobilized the same as CFA + EA group. PWTs and paw volume of rats were measured before (0 h) and at 1, 3, 5, 7, 25, and 73 h after CFA or saline injection (Figure 1). Six rats were taken from each group at 1, 3, 5, 7, 25, and 73 h, respectively. PWTs and paw volume were measured immediately and the rats were then euthanized for spinal and brain sampling. The expression of p-p38MAPK in the descending facilitatory pathway relevant areas of PAG, RVM, and SCDH was detected with immunohistochemistry.

### 2.3. Electroacupuncture

EA stimulation was conducted (9:00 a.m.), according to a previous report [30, 31]. The sets of bilateral ST36 and BL60 which has shown a good effect on inflammatory pain were selected [28]. Four stainless steel acupuncture needles (0.30 mm in diameter, 13 mm in length) were inserted into bilateral ST36 (4 mm lateral to the anterior tubercle of the tibia) and BL60 (at the ankle joint level and between the tip of the external malleolus and tendo calcaneus) acupoints. The needles in each set of EA groups were connected by a pair of wires to one output of WQ-6F Electronic Acupunctoscope (Beijing Xindonghua Electronic Instrument Co., Ltd., Beijing, China). The stimuli were set as square wave current output, with intensities in the range of 1-2 mA, 100 Hz, and 2 Hz alternating frequencies (dense and disperse mode) for 30 min [32]. Throughout the EA, rats were kept in the housing facilities without anesthesia.

### 2.4. Behavioral Test

PWT was used to assess the inflammatory pain. Its assessment was conducted immediately before (0 h) and at 1, 3, 5, 7, 25, and 73 h after CFA or saline injection. Rats were first acclimatized in individual plastic enclosures (12 × 22 × 18 cm^3^) on a metal mesh stand for 10 min before behavioral testing. The mechanical stimulus was delivered to the plantar surface of the left hind paw from the bottom floor of the plastic enclosures by an electronic von Frey anesthesiometer (ZS-Dichuang Science and Technology Development Co., Ltd., Beijing, China). A force transduction fitted with a 0.5 mm diameter polypropylene rigid tip was applied perpendicularly against the hind paw with an ever-increasing force from 0 to 50 g for a 20 s period. When the animal withdrew its hind paw, the mechanical stimulus was automatically stopped, and the force at which the animal withdrew its paw was recorded as PWT by the anesthesiometer. The procedure was repeated three times with 5 min interval. The percentage change in PWT was calculated as the following formula: Δ% = (*V*_*n*_ − *V*_0_)/*V*_0_ × 100%, where *V*_*n*_ is the PTW after CFA or saline injection and *V*_0_ is basal PWT.

### 2.5. Measurement of the Hind Paw Edema

The paw volume was assessed immediately after each PWT testing (the first test was regarded as basal paw volume) on left paw. A water displacement plethysmometer (YLS-7C, ZS-Dichuang Science and Technology Development Co., Ltd., Beijing, China) was used to measure the paw volume. The hind paw was immersed in a chamber containing distilled water up to the boundary between hairy and nonhairy skin, and the volume displacement was determined electronically. The percentage change in paw edema was calculated as the following formula: Δ% = (*T*_*n*_ − *T*_0_)/*T*_0_ × 100%, where *T*_*n*_ is the paw volume after CFA or saline injection and *T*_0_ is basal paw volume.

### 2.6. Immunohistochemistry

**Figure 2:**
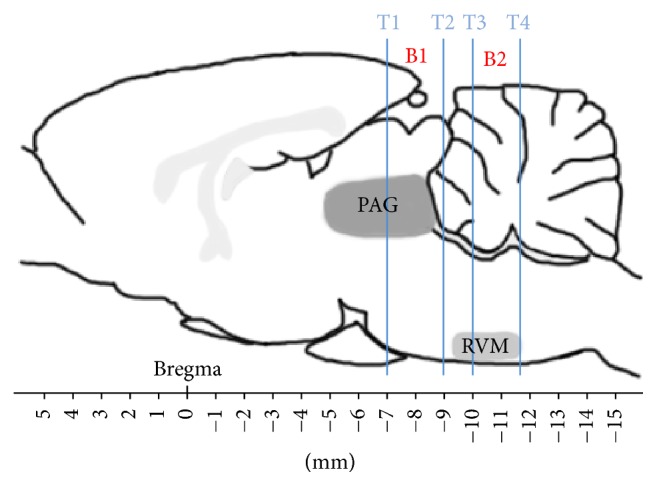
Brain sectioning. T1, T2, T3, and T4: marker tubes. T1, T2, T3, and T4 show transverse planes at −7.00 mm, −9.00 mm, −10.00 mm, and −11.60 mm rostral to the transverse plane of bregma, respectively. The locations of periaqueductal gray (PAG) and the rostral ventromedial medulla (RVM) in the brain blocks are presented. B1: a brain block containing PAG. B2: a brain block containing RVM.

SABC immunohistochemistry was employed to detect the expression level of p-p38MAPK. Rats were deeply anesthetized with sodium pentobarbital (80 mg/kg, i.p.) and were perfused transcardially with 100 mL of saline followed by 500 mL of 4% paraformaldehyde in 0.1 mol/L phosphate buffer at pH 7.4. Then, the heads of the rats were severed from the bodies and mounted in a stereotaxic instrument with the mouth bar set at the atlas standard (−3.3 mm) for blocking. According to the brain atlas of rats [33], four stainless steel marker tubes (0.8 mm in diameter) were inserted perpendicularly into the brain along the midsagittal plane of the skull by means of stereotaxic electrode carriers at −7.00 mm, −9.00 mm, −10.00 mm, and −11.60 mm from bregma, respectively (Figure 2). The brains were immersed in 4% paraformaldehyde for 48 h and then were removed from the skull and divided into several blocks according to the marker tubes. Two blocks (B1 and B2) and L4–L6 segments of spinal cord were obtained and embedded in paraffin. Each of the paraffin blocks was consecutively sectioned with a thickness of 5 *μ*m. Four slides of the PAG, RVM, and SCDH were mounted on poly-lysine coated slides, deparaffinized, and rehydrated sequentially. Among these four slides, three of them were incubated with primary antibody solution containing p-p38MAPK antibody (1:1600, CST, USA) while one was incubated with TBST instead of the antibody as negative control. The remaining experimental procedures of SABC immunohistochemistry were carried out according to the instructions (Wuhan Boster Biological Technology Ltd., Wuhan, China). The nucleus of positive cells was stained as brown yellow.

**Figure 3:**
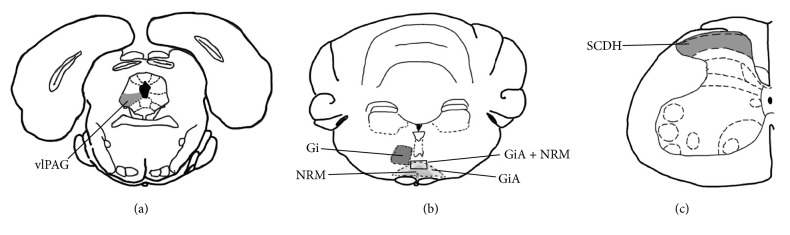
The nuclei (areas) locations used for the Phosphor-p38MAPK-Immunoreactivity cells counts. (a) The ventrolateral periaqueductal gray (vlPAG). (b) The gigantocellular reticular nucleus (Gi) and the nucleus raphe magnus + gigantocellular reticular nucleus pars alpha (GiA + NRM). (c) The spinal cord dorsal horn (SCDH).

The gigantocellular reticular nucleus (Gi), the gigantocellular reticular nucleus pars alpha (GiA), and the nucleus raphe magnus (NRM) are main part of RVM. GiA and NRM were counted together because of the difficulty to distinguish them. Therefore, p-p38MAPK-IR cells of Gi and GiA + NRM were counted to represent those of RVM. Optical images of the stained nuclei were obtained under a microscope (Nikon ECLIPSE 80I, Nikon Corporation, and Tokyo, Japan) connected to a video-based and computer-linked system (high-resolution pathological image analysis system-1000, Wuhan Qianping Ltd., Wuhan, China). Three slides of vlPAG, Gi, GiA + NRM, and SCDH were observed with 200x magnification and their locations were shown in Figure 3. The number of p-p38MAPK-IR cells on each nucleus was counted by the Image-Pro Plus 6.0 system (MediaCybernetics, Inc., Bethesda, MD, USA). The mean values calculated from each nucleus represented the p-p38MAPK per rat.

### 2.7. Statistical Analysis

PWT, paw volume, and the number of p-p38MAPK-IR cells were assessed or quantified by a skilled person who was blinded to the rat assignments. The results of PWT and paw volume were presented as the % changes ± SD. The results of the number of p-p38MAPK-IR cells were presented as the mean ± SD. The data analysis was performed with SPSS 21.0 software (SPSS Inc., Chicago, USA). The percentage changes in PWT and paw volume were analyzed with repeated measures ANOVA. The number of p-p38MAPK-IR cells was assessed using one-way ANOVA. Bonferroni's post hoc test was applied when significant differences were found. *p* < 0.05 was considered statistically significant.

## 3. Results

### 3.1. Effect of EA on Ipsilateral PWTs

**Figure 4:**
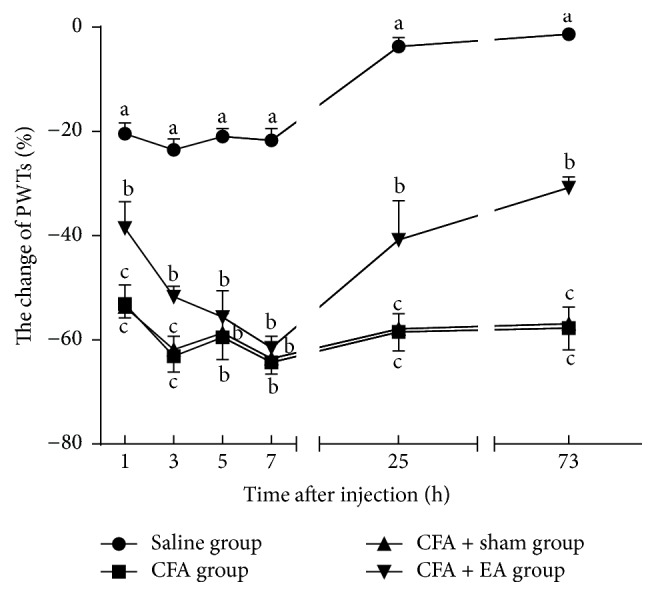
Effect of EA on ipsilateral PWTs at different time points. Values are mean ± SD, %, *n* = 6/group. The values with different letters differ significantly in the same time point (*p* < 0.01).

The results showed that PWTs in saline group decreased slightly at 1–7 h and then recovered near baseline at 25 and 73 h. PWTs of rats in CFA and CFA + EA groups were decreased at 1, 3, and 7 h obviously and reached the lowest level at 7 h. PWTs of CFA or CFA + EA group were obviously lower (*p* < 0.01) than those of saline group at 1, 3, 5, 7, 25, and 73 after CFA injection. However, PWTs of rats in CFA + EA group increased (*p* < 0.01) as compared with CFA at 1, 3, 25, and 73 h. No differences (*p* = 1.000) were found between the CFA and the CFA + sham groups in PWTs (Figure 4).

### 3.2. Effect of EA on Ipsilateral Paw Edema

**Figure 5:**
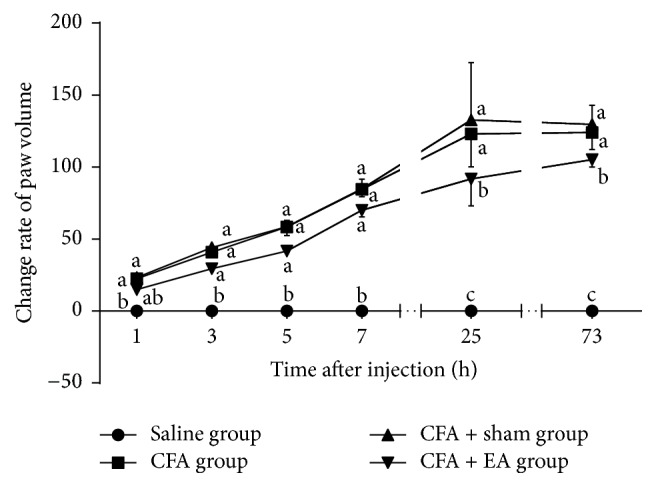
Effect of EA on ipsilateral paw edema at different time points. Values are mean ± SD, %, *n* = 6/group. The values with different letters differ significantly in the same time point (*p* < 0.05).

There were no changes of the paw volume in saline group at all measured points. The paw volume of CFA and CFA + EA groups increased gradually within 1–25 h. The paw volume of CFA group was higher (*p* < 0.01) than that in saline group during the experiment. The paw volume of CFA + EA group was higher (*p* < 0.01) than that in saline group within 3–73 h. The paw edema in CFA + EA group decreased (*p* < 0.05) at 25 and 73 h as compared with CFA group. No differences (*p* = 1.000) were detected between CFA and CFA + sham groups in the paw edema (Figure 5).

### 3.3. Phosphor-p38MAPK-Immunoreactivity

**Figure 6:**
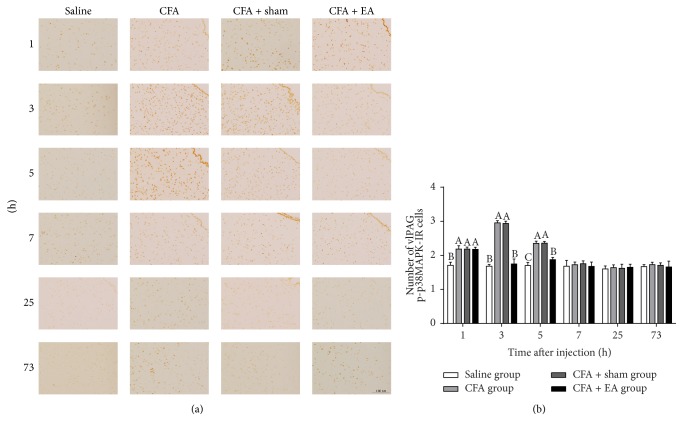
Integrated immunoreactivity analysis of p38 mitogen-activated protein kinase (p38MAPK) in the ventrolateral periaqueductal gray (vlPAG). (a) Immunohistochemical staining of Phosphor-p38MAPK-Immunoreactivity (p-p38MAPK-IR) cells in vlPAG was shown in saline, CFA, CFA + sham, and CFA + EA groups at different time points. (b) Quantification of p-p38MAPK-IR cells showing that EA suppressed expression of p-p38MAPK-IR cells in vlPAG. Values are mean ± SD, number × 10^−2^/field, *n* = 6/group. The values with different letters differ significantly in the same time point (*p* < 0.05). Scale bar represents 100 *μ*m.

Phosphor-p38MAPK-IR cells were observed in the vlPAG (Figure 6). Compared with saline group, CFA group induced an increase of p-p38MAPK-IR cells at 1–5 h (*p* < 0.01), while CFA + EA group induced an increase of p-p38MAPK-IR cells at 1 and 5 h (*p* < 0.05). After EA stimulation, p-p38MAPK-IR cells in CFA + EA group decreased (*p* < 0.01) as compared with CFA group at 3 and 5 h. There was no difference (*p* = 1.000) in the number of p-p38MAPK-IR cells between CFA and CFA + sham groups.

**Figure 7:**
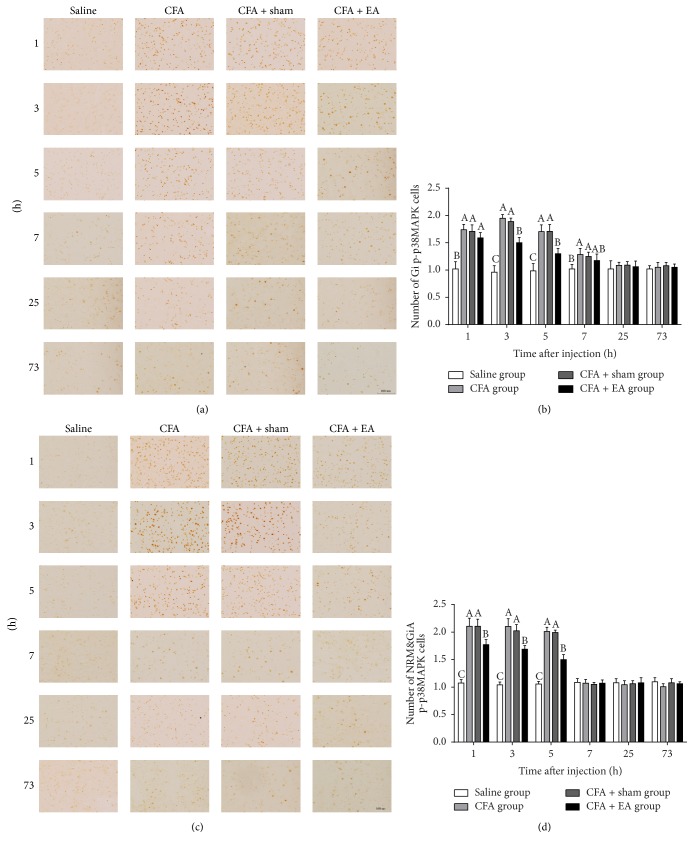
Integrated immunoreactivity analysis of p38 mitogen-activated protein kinase (p38MAPK) in the gigantocellular reticular nucleus (Gi) and the gigantocellular reticular nucleus pars alpha and the nucleus raphe magnus (GiA + NRM). Immunohistochemical staining of Phosphor-p38MAPK-Immunoreactivity (p-p38MAPK-IR) cells in Gi (a) and GiA + NRM (c) were shown in the saline, CFA, CFA + sham, and CFA + EA groups at different time points. Quantification of p-p38MAPK-IR cells showing that EA suppressed expression of p-p38MAPK-IR cells in Gi (b) and GiA + NRM (d). Values are mean ± SD, number × 10^−2^/field, *n* = 6/group. The values with different letters differ significantly in the same time point (*p* < 0.05). Scale bar represents 100 *μ*m.

The rostroventromedial medulla represented both Gi and GiA + NRM (Figure 7). Phosphor-p38MAPK-IR cells in CFA group increased (*p* < 0.01) as compared with saline group in Gi at 1–7 h or GiA + NRM at 1–5 h. In CFA + EA group, p-p38MAPK cells increased (*p* < 0.01) more than saline group in Gi and GiA + NRM at 1–5 h. After EA stimulation, p-p38MAPK cells in CFA + EA group decreased (*p* < 0.01) as compared with CFA group in GI at 3 and 5 h or GiA + NRM at 1–5 h. There was no difference (*p* = 1.000) in the number of p-p38-MAPK-IR cells between the CFA and CFA + sham groups.

**Figure 8:**
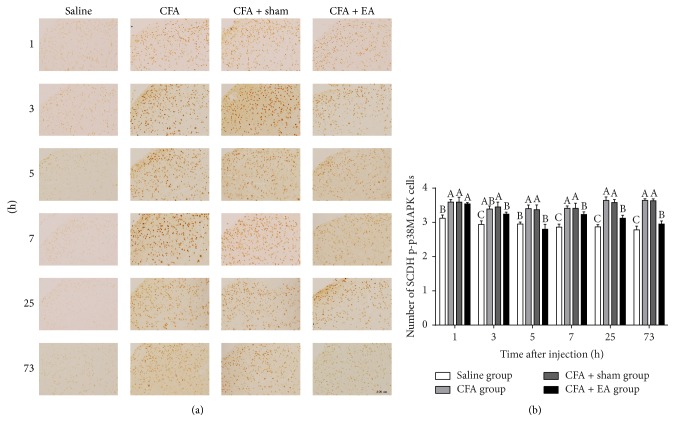
Integrated immunoreactivity analysis of p38 mitogen-activated protein kinase (p38MAPK) in the spinal cord dorsal horn (SCDH). (a) Immunohistochemical staining of Phosphor-p38MAPK-Immunoreactivity (p-p38MAPK-IR) cells in SCDH was shown in the saline, CFA, CFA + sham, and CFA + EA groups at different time points. (b) Quantification of p-p38MAPK-IR cells showing that EA suppressed expression of p-p38MAPK-IR cells in SCDH. Values are mean ± SD, number × 10^−2^/field, *n* = 6/group. The values with different letters differ significantly in the same time point (*p* < 0.05). Scale bar represents 100 *μ*m.

Phosphor-p38MAPK-IR cells were observed in the SCDH (Figure 8). Compared with saline group, CFA group induced an increase (*p* < 0.01) in the number of p-p38MAPK-IR cells in the measured points. In CFA + EA group, p-p38MAPK cells increased (*p* < 0.05) more than that in saline group at 1, 3, 7, 25, and 73 h. After EA stimulation, p-p38MAPK-IR cells in CFA + EA group decreased (*p* < 0.05) as compared with CFA group at 5–73 h. No differences (*p* = 1.000) were detected between the CFA and the CFA + sham groups in the number of p-p38MAPK-IR cells.

## 4. Discussion

CFA inflammation model was widely used for investigating the pain therapy. After the injection of CFA into paw, rats showed mechanical hyperalgesia and allodynia in 2 min and persisted for 7–14 d [34, 35], while paw edema started in 10 min and rose to peak at 3 d [35–38]. In the present study, the pain threshold was decreased at 1 h and still remained at a low level in 73 h after CFA injection, while CFA-induced inflammatory paw edema increased significantly and remained at high level during the whole experiment. These results showed the same tendency of inflammatory symptoms with previous studies.

Acupuncture therapy, especially EA, can induce potent analgesia to relieve persistent pain. Han [39] reported that frequency-dependent EA analgesia is mediated by the different opioid receptor subtypes. A frequency of 2/100 Hz EA simultaneously activating *μ*-, *δ*-, and *κ*-opioid receptors was reported to induce more effective analgesia than a single frequency (2 Hz, 15 Hz, or 100 Hz) in rats [40]. Acupoints and EA times are also important factors associating with EA-induced anti-inflammatory pain effect. ST36 acupoint has been widely used to induce analgesic effect in rats in different pain model as well as BL60 acupoint [32, 41, 42]. In CFA model, EA applied at the bilateral ST36 and BL60 acupoints once per day could induce an increase in the PWTs without affecting the paw volume for 3 days [43]. Fang et al. [44] also reported that EA at ST36 and BL60 relieved CFA-induced inflammatory pain at 6, 25, and 49 h after CFA injection. Similar results were confirmed in a report that EA at ST36 and BL60 once per day could increase mechanical allodynia at 3 and 14 d and decreased paw edema at 14 d after CFA injection [28]. In the present study, the PWTs of CFA + EA group rats (receiving 2 Hz and 100 Hz alternating frequencies of EA at ST36 and BL60 acupoints for 30 min, once per day) increased gradually and were higher than CFA group at 1, 3, 25, and 73 h after CFA injection. As EA repeated, its effect increased compared with CFA control group. Although EA lasted for 30 min each time, EA effect at 25 h was obviously higher than that at 5 h and was lower than that at 73 h within the EA group. These results suggested that EA-induced anti-inflammatory pain exhibited immediate and cumulative effects. In addition, the paw edema in CFA + EA group markedly decreased at 25 and 73 h as compared with CFA group (*p* < 0.05), indicating that EA exerts some therapeutic effect on inflammation.

Numerous studies indicated that p38MAPK participated in inflammatory response in central nerve system. In CFA model, the number of p-p38MAPK-IR cells was significantly increased in NRM for 0.5–1 h and in GiA at 0.5, 3, and 5 h [17]. Fang et al. [29] illustrated that CFA induced an increasing number of spinal p-p38MAPK-IR cells at 3, 7, and 14 d. Our results showed CFA induced the increasing p-p38MAPK-IR cells in the PAG and RVM, rising to a peak during 1–3 h and then recovering back at 5–7 h after the injection of CFA, while the number of p-p38MAPK-IR cells in SCDH increased lasting for 73 h. Moreover, several researches provided evidence that EA exert anti-inflammatory pain by inhibiting the activation of p38MAPK in spinal level. Yi et al. [28] applied CFA into rat paw and found CFA-induced increasing of p-p38MAPK-IR cells was suppressed by EA at 3 and 14 d. The same results were found in Fang et al.'s study [29] that EA attenuated CFA-induced inflammation pain and declined the level of spinal p-p38MAPK at 14 days after EA application. Currently, EA decreased the number of p-p38MAPK-IR cells in GiA and PAG at 3 and 5 h and in Gi + NRM at 1–5 h and declined the activation of spinal p38MAPK at 3–73 h after CFA injection. The results suggest EA could alleviate mechanical hyperalgesia and inhibited the expression of p-p38MAPK in PAG-RVM-SCDH.

As activated p38MAPK in spinal and supraspinal sites promotes the production of inflammatory mediators, it is possible that activated p38MAPK in the PAG, RVM, and SCDH can increase descending pain facilitation. Accordingly, disruption of p38MAPK or p38MAPK downstream inflammatory mediators might reverse behavioral hyperalgesia and allodynia. At supraspinal level, p-p38MAPK was localized and increased in PAG after chronic constriction nerve injury [16] and in RVM after carrageenan-induced inflammation [17]. Pretreatment with microinjection of p38MAPK inhibitor SB203580 in RVM produced a significant attenuation of behavioral hypersensitivity resulting from hind paw inflammation [9]. In the present study, EA induced anti-inflammatory effect and decreased CFA-increased expression of p-p38MAPK in PAG and RVM. In addition, p38MAPK as promoter of the interleukin-1*β* (IL-1*β*) could lead to the activation of IL-1*β* receptors [45]. Previous reports indicated that increased IL-1*β* in PAG partly mediated and enhanced pain facilitation [46]. Further, once IL-1*β* synthesis was inhibited in PAG, mechanical allodynia and thermal hyperalgesia were significantly attenuated [46]. In a carrageenan model of inflammatory pain, EA administrated at ST36 and BL60 significantly inhibited thermal hyperalgesia and carrageenan-increased mRNA expression of IL-1 receptor type I in PAG [47]. Taken together, these studies suggest that EA could attenuate inflammatory pain by disrupting activation of p38MAPK or p38MAPK downstream inflammatory mediators in RVM and PAG.

At spinal level, the expression level of p-p38MAPK increased after peripheral inflammation which is induced by CFA [48], bee-venom [49], capsaicin [50], or formalin [20, 51]. Further, p38 inhibitor (SB20358 or SD-282) was shown to effectively attenuate hyperalgesia in peripheral tissue inflammation (intraplantar formalin or carrageenan) [20]. Fang et al. [29] found that EA at ST36 and BL60 acupoints could reduce the numbers of p-p38MAPK-IR cells in SCDH in CFA rats, and it was consistent with the present study. In addition, activated p38MAPK upregulated the synthesis of several inflammatory mediators such as cyclooxygenase-2 (COX-2), IL-1*β*, and tumor necrosis factor-*α* (TNF*α*) in the spinal cord to enhance central sensitization [20, 45], whereas these inflammatory mediators could be blocked by pretreatment with intrathecal injection of p38MAPK inhibitors [14, 52, 53]. In CFA-induced inflammatory pain model, EA could alleviate inflammatory pain behavior by downregulated COX-2 in the spinal cord [43]. CFA-increased spinal IL-1*β* and TNF-*α* were attenuated following EA treatment [54]. Taken together, these results indicate that EA may induce antihyperalgesic effect through suppressing p-p38MAPK and its downstream inflammatory mediators in SCDH in a complex mechanism.

## 5. Conclusion

EA could significantly inhibit CFA-induced decline of PWTs and CFA-induced increase of paw edema. The number of p-p38MAPK-IR cells increased in PAG, RVM, and SCDH after CFA injection, while it reduced after EA application. These results suggested that EA could disrupt descending facilitation in PAG, RVM, and SCDH and exhibits antihyperalgesic effect by inhibiting p38MAPK activation in descending facilitatory pathway.

## Abbreviations

CFA: The complete Freund adjuvant
COX-2: Cyclooxygenase-2
EA: Electroacupuncture
Gi: The gigantocellular reticular nucleus
GiA: The gigantocellular reticular nucleus pars alpha
IL-1: Interleukin-1
MAPK: Mitogen-activated protein kinase
NRM: The nucleus raphe magnus
PAG: The periaqueductal gray
p-p38MAPK: Phosphorylated p38MAPK
p-p38MAPK-IR: Phosphor-p38MAPK-Immunoreactivity
PWT: Paw withdrawal threshold
RVM: Rostral ventromedial medulla
SCDH: Spinal cord dorsal horn
vlPAG: Ventrolateral periaqueductal gray.
